# Change of pre-loss grief in relatives of cancer patients: a longitudinal study

**DOI:** 10.3389/fpsyg.2026.1880352

**Published:** 2026-07-16

**Authors:** Viktoria Schmidt, Julia Treml, Julia Kaiser, Anette Kersting

**Affiliations:** Faculty of Medicine, University of Leipzig, Leipzig, Germany

**Keywords:** anticipatory grief, bereavement, cancer, pre-loss grief, support seeking

## Abstract

**Introduction:**

Before the loss of a loved one to cancer, many relatives experience feelings of pre-loss grief. While pre-loss grief seems to have adverse health impacts, its longitudinal associations are still unclear. Therefore, this study aims to investigate the change of pre-loss grief over time, predictors of change and its relationship with psychological health over a time period of 1.5 years.

**Methods:**

Fifty-two relatives of people with cancer who were at least 18 years old and spoke German were included. Multiple linear regression analysis and paired *t*-tests were performed.

**Results:**

The total pre-loss grief score significantly decreased over time (*p* = 0.009), while at the subscale level only emotional pain showed a significant decrease. Attendance of a psychotherapist (*β* = −0.452, *p* = 0.041) more than once per month compared to never, less than or once per month was associated with a greater reduction in pre-loss grief. While psychological health did not predict later pre-loss grief (*p* > 0.05), pre-loss grief was found to predict higher levels of anxiety (*β* = 0.335, *p* = 0.046) 1.5 years later.

**Discussion:**

Findings suggest that emotional pain may lessen over time, whereas other dimensions of pre-loss grief may remain stable. At the same time, because pre-loss grief is associated with some adverse health outcomes 1.5 years later, relatives with high levels of pre-loss grief may need additional support. Furthermore, this study provides preliminary insights into helpful intervention approaches for pre-loss grief. To understand underlying mechanisms, further dismantling studies are needed.

## 1 Introduction

Nearly one in six deaths can be attributed to cancer, causing almost 10 million deaths in 2020 (WHO, 2020). The World Health Organization further predicts that this number can rise up to 16.2 million deaths by the year 2040 (Schüz and Espina, 2021). While an early diagnosis can increase the chance for survival (Ginsburg et al., 2020), some cases still remain incurable and palliative care is needed. For relatives, such illness trajectories may involve an ongoing awareness of possible loss even before the patient’s death. Many individuals with cancer prefer to be cared for at home (Higginson and Sen-Gupta, 2000; Gomes et al., 2013). Similarly, often family members prefer an at-home care (Woodman et al., 2016). Therefore, there are millions of caregivers of people with cancer worldwide, although the exact number is unknown and in the United States alone was 2.8 million in the year 2020 (National Alliance for Caregiving, 2020).

Family members of individuals with cancer often experience high levels of caregiver burden and show elevated risk for psychological disorders and low physical health (Mosher et al., 2013; Trevino et al., 2018; Karimi Moghaddam et al., 2023). Beyond caregiving burden, relatives of people with cancer may also experience grief-related responses even before the death occurs. The occurrence of grief before a loss is described as pre-loss grief in the literature and can be defined as a “grief reaction due to multiple losses during end-of-life caregiving” (Nielsen et al., 2017b, p. 2048). These losses may relate to loss of shared time and future between the caregiver and loved one, to the inevitable death and everyday life changes (Nielsen et al., 2017b). While pre-loss grief has often been examined in relatives or caregivers of individuals in end-of-life care, it is measured heterogeneously (Treml et al., 2021). It therefore may not only focus on the death and future without a loved one, but also refer to grief reactions associated with present changes and losses due to the ongoing illness (Singer et al., 2022a). Given the varying definitions of grief prior to a loss, and in order to include participants who may experience grief due to the ongoing illness, this study adopted a broader definition of pre-loss grief and included all relatives of people with cancer, regardless of disease stage or treatment situation. Consequently, the construct assessed in the present study may reflect grief-related distress in the context of cancer more broadly, rather than exclusively in the context of advanced illness, palliative care or end-of-life caregiving. Despite these conceptual differences, pre-loss grief appears to affect a substantial proportion of relatives. A recent meta-analysis estimated the overall prevalence for pre-loss grief in relatives of loved ones suffering from a life-threatening illness at 24.8%, indicating a high percentage of affected relatives (Kustanti et al., 2022). Higher pre-loss grief levels appear to be associated with poor mental health in caregivers, as a positive association with depression and anxiety symptoms prior to and after losing a loved one has been found (Treml et al., 2021). However, this relationship has often been examined cross-sectionally (Tomarken et al., 2008; e.g., Nielsen et al., 2017b). Moreover, longitudinal studies have investigated this relationship mostly in a unidirectional way, without considering whether mental health may be predictive of pre-loss grief (Coelho et al., 2017; Nielsen et al., 2017a). For example, studies investigating grief in relatives after the loss of a loved one found that not only grief significantly predicted depression, but also that depression predicted grief 12 months later (Prigerson et al., 1996). However, it remains unclear, if this may also be true for pre-loss grief. Nevertheless, findings from some studies indicate that aspects of psychological health, such as a history of depression, may also predict subsequent levels of pre-loss grief, pointing to possible bidirectional associations. (Tomarken et al., 2008).

Furthermore, while much research has been done in the area of correlates of pre-loss grief and its negative association with post-loss adjustment (e.g., Nielsen et al., 2016; Treml et al., 2021), little is known about the progression of pre-loss grief over time. Nevertheless, advances in cancer treatment may result in a long time between the cancer diagnosis and the loss of a loved one, (Lage and Crombet, 2011). Thus, feelings of pre-loss grief may persist for an extended period of time, possibly changing over time. Conceptually, pre-loss grief may change over time because relatives’ experiences of illness progression, uncertainty, and care-related demands may change during the trajectory of the illness, which influences how pre-loss grief is experienced (Fee et al., 2021). Post-loss grief has been found to decrease over time for most people, with 3–4% of bereaved developing a prolonged grief disorder- a persistent grief reaction causing functional impairment (Jordan and Litz, 2014; Rosner et al., 2021). However, it is unclear whether this overall decrease in grief symptoms might also be true for pre-loss grief. Studies on the progression of grief usually examine different trajectories of grief after a loss (Sveen et al., 2018; Djelantik et al., 2022), or the change from pre-loss grief to post-loss grief (Nielsen et al., 2019). For example, Nielsen et al. (2019) studied different grief trajectories that included grief both before and after a loss. However, Nielsen et al. (2019) included only one measurement time for pre-loss grief, not investigating how feelings of grief may change over time even before losing a loved one. There appears to be only two studies that have investigated the change of pre-loss grief over time with ambivalent results (Singer et al., 2022b). While Holm et al., (2020) did not find any change in pre-loss grief over time, Singer et al. (2022b) found a significant decrease in pre-loss grief at the second assessment, while the time between the two measurement points was relatively short (1 month, Singer et al., 2022b), so that associations with longer-lasting loss processes were not investigated. Thus, studies on the change of pre-loss grief are needed.

Similarly, there is little knowledge on what might be helpful to reduce pre-loss grief over time. Existing evidence is largely based on grief after loss, where the attendance of a support group has been described as beneficial by participants, and was found to predict reductions in grief symptoms (Ghesquiere et al., 2013; Robinson and Pond, 2019). Support groups may also benefit relatives during the end of life of a loved one (Henriksson et al., 2013), although their specific association with pre-loss grief reduction has not been studied yet. Evidence on counseling similarly suggests perceived benefits before and after a loss (Gamino et al., 2009; Dhiliwal and Muckaden, 2015), without quantitatively assessing its impact on the reduction of grief severity. Even less is known about psychiatric care, which some relatives use before a loss (Gamino et al., 2009). In contrast, visits to a general practitioner or family doctor was found to not effectively reduce grief symptoms after a loss (Ghesquiere et al., 2013).

On the other hand, psychotherapy, especially cognitive-behavioral approaches, have been found to be effective in reducing grief symptoms after a loss (Boelen and Kolchinska, 2022). For pre-loss grief, there are limited intervention studies in which various approaches have proven to effectively reduce pre-loss grief, e.g., a family-based dignity intervention or pre-loss group therapy (Ott et al., 2010; Supiano et al., 2021; e.g., Ghezeljeh et al., 2023). However, the literature on this topic is limited. Moreover, social support has found to be negatively associated with grief symptoms before and after a loss (Callahan, 2000; Tomarken et al., 2008), however its’ direct influence on the change of pre-loss grief over time is unclear. Due to the lack of studies on the effects of different types of support for people suffering from pre-loss grief, it is necessary to further investigate how these different opportunities for support may help reduce grief symptoms over time. This seems particularly important due to the adverse health impacts of high levels of grief before and after a loss (Simon, 2013).

To address the aforementioned gaps in the literature, this study aims to investigate pre-loss grief in a longitudinal study design. Given the limited and heterogeneous evidence, the study was exploratory and no directional hypotheses were formulated. Specifically, the research aims were to investigate:

1. Change of pre-loss grief over time.
2. The relationship between the attendance of various types of support and change in pre-loss grief over time and.
3. The relationship between pre-loss grief and psychological health in relatives of people with cancer.

## 2 Materials and methods

### 2.1 Study procedure

The study was conducted according to the Declaration of Helsinki and was approved by the local Ethics Committee (Ethics Committee of the Medical Faculty of the University of Leipzig, reference number 046/20-ek). Participants were recruited from August 2020 to September 2021 for the first time point (T1). The follow-up assessment was conducted between February 2022 and March 2023–1.5 years after the first assessment (T2).

Participants were mainly recruited through social media-via Facebook groups and social media networks. In addition, hospices and other health care services such as counseling centers were contacted. Convenient sampling was used.

Study information was linked online and interested individuals could start the survey by following a link. Participants provided written informed consent by confirming a consent form on the first page of the survey.

### 2.2 Participants

As conceptualizations of pre-loss grief differ across the literature, the present study used a broad operationalization of the construct and therefore included all relatives of people with cancer, irrespective of disease stage or treatment situation. Inclusion criteria were: (a) a minimum age of 18 years, (b) fluency in the German language, (c) being a relative of a person with cancer and (d) digital informed consent. In total, 299 participants completed the first assessment. Participants had the option to choose an anonymous participation or providing their contact information for a follow-up study. Of the 299 participants, 126 provided their contact information. Of these, 88 (69.8%) participated in the second survey, with 36 indicating that their loved one had already passed away. Thus, the final sample consisted of 52 people where the loved one was still alive and thus pre-loss grief could be assessed. Participants whose loved one had died by the second assessment were excluded from the longitudinal analyses, as pre-loss grief was no longer assessable after the death and post-loss grief was assessed using a different measure.

### 2.3 Measures

At the first assessment point, demographic characteristics were assed via self-constructed self-report questions, e.g., regarding the age and gender of the participants and their loved one. Participants were also asked how often they had contact to (a) a Support Group, (b) a Counselor, (c) a psychiatrist, (d) a General Practitioner or Specialist, (e) psychotherapist or (d) Other individuals or groups because of the psychological stress due to the illness of the loved one (1 never, less than or once per month, 2 = more than once per month). Additionally, the subjective prognosis of the loved one was assessed via the question: “How likely do you think the person with cancer will die within the next 5 years? Choose a point on the scale between 0 and 100%.”

Social support was assessed at the first assessment point using the ENRICHD Social Support Instrument (ESSI, Kendel et al., 2011). The scale contains five items with a 5-point Likert scale (1 = never, 5 = always). Items can be summed up to a total score and higher values indicate higher social support. The internal consistency for the total scale was good in this study (*α* = 0.89).

Several scales were measured at the first and second assessment point:

Pre-loss grief was measured via the Caregiver Grief Scale (Meichsner et al., 2016). This scale contains 11 items, which can be answered on a 5-point Likert-scale (1 = strongly disagree; 5 = strongly agree). The items can be summed up to a total sum score, or to the following four subscales: emotional pain (items 1–3), relational loss (items 4–6), absolute loss (items 7–9) and acceptance of the loss (items 10 and 11). A higher score in the total scale or in the subscales implies higher grief. Cronbach’s alpha for the total scale was acceptable-good in this study (α_T1_ = 0.75, α_T2_ = 0.84). Given that the present study included relatives of people with cancer across different sages and treatment situations, the Caregiver Grief Scale was selected rather than instruments more closely linked to prolonged grief concepts, which have been mainly applied in end-of-life caregiving contexts. Although the Caregiver Grief Scale was originally developed and validated in dementia caregivers, it has also been psychometrically validated in a broader caregiver sample that included caregivers of patients with cancer, although authors favored a one-factor solution (Vasconcelos-Raposo et al., 2019).

Depression was measured via the Patient-Health Questionnaire (PHQ-9, Kroenke et al., 2001). The scale contains nine items with a 4-point Likert scale (0 = not at all, 3 = nearly every day). A higher sum score of all items represents a higher depression severity. Cronbach’s Alpha was good to excellent in this study for the total scale (*α*_T1_ = 0.90, α_T2_ = 0.89).

Anxiety was assessed using the Generalized Anxiety Disorder Scale (GAD-7, Spitzer et al., 2006) containing seven questions with a 4-point Likert scale (0 = not at all, 3 = nearly every day). A higher sum score represents higher anxiety severity. Cronbach’s Alpha was good to excellent in this study (α_T1_ = 0.91, α_T2_ = 0.88).

Lastly, somatization was measured via the Patient Health Questionnaire (PHQ-15, Kroenke et al., 2002). The scale contains 15 items and a 4-point Likert scale was used (0 = not at all, 4 = nearly every day). A higher total sum score represents higher severity in somatic symptoms. Internal consistency was acceptable in this study (α_T1_ = 0.79, α_T2_ = 0.75). As no formal *a priori* power analysis was conducted prior to data collection, sensitivity power analyses were calculated in G*Power to contextualize the statistical power of the final longitudinal samples.

### 2.4 Statistical analysis

Statistical analysis was performed using the Statistical Package for Social Sciences, version 29 (IBM®SPSS®). The significance level was set to α = 0.05.

To examine change of pre-loss grief over time, paired t-tests comparing the total scale and the subscales of pre-loss grief between the two time points were performed.

To investigate predictors of change in pre-loss grief, multiple linear regression analysis with absolute change scores of pre-loss grief (T2-T1, see Bryant et al., 2017) as the dependent variable was performed. Social support and contact to various individuals or groups (e.g., support group, psychiatrist,…) at the first assessment point (T1) were added as independent variables, controlling for age, gender, pre-loss grief and subjective prognosis at T1. In addition, independent-samples t-tests were conducted to examine baseline differences in pre-loss grief, depression, anxiety, and somatization between participants who used each type of support more than once per month and those who did not.

Moreover, to examine the direction of the relationship between pre-loss grief and psychological health, multiple linear regressions were performed (controlled for age, gender,subjective prognosis and baseline distress (measured with pre-loss grief, depression, anxiety or somatization, depending on the respective models at T1): (a) with depression, anxiety, somatization (T1) predicting pre-loss grief (T2) and (b) with pre-loss grief (T1) predicting depression, anxiety and somatization (T2). For each analysis, only participants with complete data sets were included. Therefore, sample sizes varied slightly across analyses because missing values differed across the variables included in each analysis.

Lastly, completers and dropouts were compared on baseline variables using independent-samples *t*-tests and χ^2^ tests or Fisher’s exact tests. Assumptions for all analyses were assessed and met. For all analyses, the Benjamini and Hochberg (1995) correction was applied.

## 3 Results

### 3.1 Preliminary analysis

Most participants were women (88.5%) with a mean age of 38.67 (SD = 12.10). The most common types of cancer were breast cancer (15.5%), leukemia (15.5%), gastrointestinal cancer (11.5%) and eye, brain or central nervous system cancer (11.5%). Most participants had a German nationality (92.3%) and a high school education (67.3%). A detailed sample description can be found in Table 1.

**Table 1:** Characteristics of the sample (*n* = 52).

Characteristics	Variable	M (SD)/*n* (%)
Characteristics of participants	Age	38.67 (12.10)
	Gender	
	Female	46 (88.5)
	Male	6 (11.5)
	School education	
	Low	1 (1.9)
	Medium	16 (30.8)
	High	35 (67.3)
	Diseased person is my	
	Parent	21 (40.4)
	Partner	13 (25.0)
	Child	8 (15.4)
	Sibling	5 (9.6)
	Friend	3 (5.8)
	Other	2 (3.8)
	Religious	36 (69.23)
	Nationality	
	German	48 (92.3)
	Other	4 (7.7)
	Cancer type	
	Oral cavity and oropharyngeal cancer	1 (1.9)
	Gastrointestinal cancer	6 (11.5)
	Lung cancer	5 (9.6)
	Melanoma	1 (1.9)
	Breast cancer	8 (15.5)
	Genital cancer	5 (9.6)
	Cancer of the urinary system	1 (1.9)
	Eye, brain and central nervous system cancer	6 (11.5)
	(Non-) Hodgkin’s lymphoma	6 (11.5)
	Leukemia	8 (15.5)
	Not known/Other	5 (9.6)
Characteristics of people with cancer	Age	47.69 (21.26)
	Gender	
	Female	28 (53.8)
	Male	24 (46.2)
Pre-loss grief	T1	3.45 (0.69)
	T2	3.02 (0.86)

### 3.2 Change of pre-loss grief over time

Pre-loss grief significantly changed between the two time points. The total pre-loss grief sore significantly decreased 1.5 years after the initial assessment (mean difference 0.435 [0.220–0.651], *p* = 0.009, see Table 2). From all subscales only emotional pain (mean difference 0.814 [95% CI 0.477–1.152], *p* = 0.009) significantly decreased over time. On the other hand, relational loss (*p* = 0.079), absolute loss (*p* = 0.388) and acceptance of loss did not significantly change over time (*p* = 0.470).

**Table 2:** Paired *t*-tests for comparison of pre-loss grief between two time points.

Variable	M of difference (95% CI)	SD	T	*df*	Significance^a^
Pre-loss grief sum scale	0.435 (0.220 to 0.651)	0.775	4.050	51	0.009
Emotional pain	0.814 (0.477 to 1.152)	1.213	4.842	51	0.009
Relational loss	0.359 (0.078 to 0.640)	1.009	2.564	51	0.079
Absolute loss	0.282 (−0.054 to 0.618)	1.205	1.688	51	0.388
Acceptance of loss	0.212 (−0.083 to 0.506)	1.059	1.441	51	0.470

a Corrected by Benjamin & Hochberg. *n* = 52.

### 3.3 Predictors of change in pre-loss grief

The regression model yielded one significant predictor of change in pre-loss grief. The attendance of a psychotherapist (*β* = −0.452, *p* = 0.041) more than once per month compared to never, less than or once per month was associated with a greater reduction in pre-loss grief 1.5 years later (see Table 3). No statistically significant baseline differences in pre-loss grief, depression, anxiety, or somatization were found between participants who used support more than once per month and those who did not, regardless of support type (all *p* > 0.05). The number of participants reporting support use more than once per month was low across support types: support group: *n* = 1; counselor: *n* = 2; psychiatrist: *n* = 1; general practitioner/specialist: *n* = 2; psychotherapist: *n* = 3; and other support: *n* = 4.

**Table 3:** Predictors of change in pre-loss grief.

Variable	Unstandardized B (95% CI)	Standard Error	*β*	*T*	Significance^c^
Intercept	0.190 (−1.647 to 2.027)	0.906		0.210	1.000
Age	0.003 (−0.016 to 0.022)	0.009	0.050	0.340	1.000
Gender^a^	0.678 (−0.101 to 1.457)	0.384	0.261	1.765	0.388
Pre-loss grief T1	−0.359 (−0.711 to −0.008)	0.173	−0.304	−2.074	0.288
Prognosis	−0.001 (−0.007 to 0.005)	0.003	−0.032	−0.236	1.000
Contact to^b^:					
Support Group	0.075 (−1.353 to 1.503)	0.704	0.013	0.106	1.000
Counselor	−1.948 (−3.427 to −0.470)	0.729	−0.351	−2.672	0.072
Psychiatrist	1.187 (−0.660 to 3.035)	0.911	0.214	1.304	0.523
General practitioner/specialist	0.285 (−0.938 to 1.508)	0.603	0.072	0.473	0.969
Psychotherapist	−1.793 (−3.000 to −0.587)	0.595	−0.452	−3.014	0.041
Other	0.603 (−0.216 to 1.422)	0.404	0.210	1.494	0.468
Social Support	−0.003 (−0.057 to 0.051)	0.027	−0.019	−0.125	1.000

a Female or diverse gender compared to male gender.

b Attended more than once per month compared to never, less than or 1 time per month for emotional stress due to the illness of the loved one.

c Corrected by Benjamin and Hochberg.

*R*^2^ = 0.442, Adjusted *R*^2^ = 0.271. *n* = 48.

### 3.4 Relationship between pre-loss grief and psychological health

A regression model showed that psychological health (depression, anxiety, somatization, controlled for age, gender, pre-loss grief and prognosis) at T1 did not significantly predict pre-loss grief at T2 (all *p* > 0.05).

On the other hand, higher levels of pre-loss grief at T1 were found to predict higher levels of anxiety (*β* = 0.335, *p* = 0.046) 1.5 years later (see Table 4). Pre-loss grief did not predict somatization (*β* = −0.047, *p* = 0.994) or depression (*β* = 0.162, *p* = 0.505), when controlling for baseline depression or somatization.

**Table 4:** Relationship between pre-loss grief and psychological health 1.5 years later.

Variable	Depression T2		Anxiety T2		Somatization T2	
	*β* (95% Cl)	Significance^a^	*β* (95% Cl)	Significance^a^	*β* (95% Cl)	Significance^a^
Intercept	(−9.446 to 6.481)	1.000	(−7.007 to 6.630)	1.000	(−1.045 to 9.544)	0.411
Age	−0.035 (−0.121 to 0.087)	1.000	−0.177 (−0.162 to 0.014)	0.388	−0.092 (−0.096 to 0.038)	0.775
Gender	0.023 (−3.424 to 4.244)	1.000	−0.121 (−5.237 to 1.511)	0.644	−0.019 (−2.811 to 2.372)	1.000
Prognosis T1	0.005 (−0.036 to 0.038)	1.000	0.075 (−0.020 to 0.042)	0.853	−0.117 (−0.037 to 0.011)	0.644
Baseline distress T1^b^	629 (0.331 to 0.746)	0.009	0.512 (0.246 to 0.664)	0.009	0.771 (0.403 to 0.776)	0.009
Pre-loss grief T1	0.162 (−0.674 to 3.430)	0.505	0.335 (0.772 to 4.210)	0.046	−0.047 (−1.587 to 1.057)	0.994
*R*^2^ (Adjusted *R*^2^)	0.526 (0.470)		0.548 (0.495)		0.545 (0.492)	

a Corrected by Benjamin and Hochberg.

b Baseline distress contained depression at T1 for predicting depression at T2, anxiety at T1 for predicting anxiety at T2 and somatization at T1 for predicting somatization at T2.

*n* = 49.

Sensitivity power analyses indicated that the available samples allowed detection of effects of dz. = 0.396 or larger for paired t-tests examining change over time (*n* = 52), *f*^2^ = 0.173 or larger for individual support predictors of change in pre-loss grief (*n* = 48), *f*^2^ = 0.168 or larger for pre-loss grief as a predictor of psychological health (*n* = 49), and *f*^2^ = 0.245 or larger for psychological health variables as predictors of pre-loss grief (*n* = 49). Moreover, dropout analyses showed no significant differences between completers and dropouts in all variables assessed (*p* > 0.05).

## 4 Discussion

This study aimed to investigate the change of pre-loss grief over time, the association of various types of support and change in pre-loss grief, as well as the relationship between pre-loss grief and psychological health in relatives of people with cancer. Results indicate that pre-loss grief decreased over a period of 1.5 years. This is in line with a previous study by Singer et al. (2022b), who found a reduction in pre-loss grief symptoms over time, whereby a relatively short period of 1 month was examined. Thus, grief symptoms could also decrease in the case of long-term cancer processes. This is similar to grief after a loss, which also shows an overall reduction in most cases (Jordan and Litz, 2014). At the same time, results found that only the subscales emotional pain decreased over time, while the other subscales (relational loss, absolute loss, acceptance of loss) remained relatively stable. Results of this study may help to contextualize previous ambivalent findings of previous studies observing either a reduction or stability in grief symptoms over time (Holm et al., 2020; Singer et al., 2022b), suggesting that only some aspects of pre-loss grief may decrease over time. Results suggest that some aspects like the emotional pain of relatives may have decreased (e.g., items for feeling terrific sadness or perceiving the situation as unacceptable in one’s heart). On the other hand, other aspects of the loss may still have been evident due to the continued presence of the ill person (e.g., items for feeling that life is empty without the loved one; or longing for what was in the past). However, as previous studies often differ in the measurement scales used for pre-loss grief (Singer et al., 2022a), results could vary depending on the employed scales, thus highlighting the need for a consistent definition and operationalization of pre-loss grief. However, this interpretation should be considered cautiously. As the decrease in the total pre-loss grief score was on the subscale level only found in emotional pain, the findings should not be interpreted as evidence for a general decrease in all dimensions of pre-loss grief. Moreover, due to the lack of detailed information on patients’ clinical course, treatment response, remission status, or disease progression, it remains unclear whether the reduction in emotional pain reflects a change in grief itself or rather reduced uncertainty, reduced perceived threat of loss, or an improvement or stabilization of the patient’s clinical condition during the follow-up period. Future studies investigating changes in pre-loss grief should therefore include more detailed information on patients’ clinical course, treatment response, and disease status.

Furthermore, this is the first study to prospectively investigate the association of attendance of various types of support on change of pre-loss grief over time. Results revealed that visiting a psychotherapist more than once per month compared to never, less than or once per month was associated with a greater decrease in pre-loss grief 1.5 years later. This is in line with previous studies demonstrating the effectiveness of psychotherapy for relatives suffering from pre-loss grief (Ott et al., 2010; Supiano et al., 2021; e.g., Ghezeljeh et al., 2023). However, since this study did not differentiate between different therapy approaches, further studies are needed to determine which treatment options for relatives with high levels of pre-loss grief may be beneficial. Moreover, as only three people attended a psychotherapist and the attendance of other support types was also very low (*n* = 1–4), findings regarding support-use variables are rather exploratory and should be interpreted cautiously. Nevertheless, literature suggests that for people at high risk of developing prolonged grief disorder after a loss, an early therapy approach focusing on pre-loss grief during the end-of-life phase appears to be beneficial for reducing the risk for developing prolonged grief disorder (Supiano et al., 2021).

The attendance of a counselor, support group, psychiatrist, general practitioner/specialist and social support were not associated with pre-loss grief reduction. However, because support use was low, findings have to be interpreted cautiously. While support groups have been found effective in reducing post-loss grief (Ghesquiere et al., 2013), associations with pre-loss grief have not yet been reported, limiting generalizability of previous studies. Moreover, most studies report on the subjective perceived positive experience in support groups without allowing quantitative conclusions to be made (Robinson and Pond, 2019), indicating the need for further studies. Also, while social support can mitigate adjustment to loss (Kreicbergs et al., 2007), informal support from close friends and family may not be sufficient to address pre-loss grief Similarly, general practitioners have also been found to have limited knowledge and training on the topic of grief, indicating the need for further training (Muhammed et al., 2023). As the association between these types of support and changes in pre-loss grief have not been examined before, and attendance of these categories was very low in this sample, further studies are necessary.

Lastly, results found that psychological health was not associated with pre-loss grief, while in turn higher levels in pre-loss grief were associated with higher levels in anxiety 1.5 years later. This is in line with previous studies finding higher levels of pre-loss grief to be associated with adverse health impacts over time (e.g., Coelho et al., 2017; Nielsen et al., 2017a). However, as pre-loss grief was not associated with depression and somatization, when controlling for baseline distress, previous findings may reflect the stability of psychological distress over time.

## 5 Limitations

There are several limitations to the study. Most participants were women (88.5%) and had a medium to high level of school education (98.1%), possibly influencing the representativeness of the sample. Future studies should try to include a higher percentage of men and people from different levels of education. Because convenience sampling was used, the findings may not generalize to all relatives of people with cancer and may be affected by self-selection bias in this psychologically sensitive context.

Moreover, as the provision of contact information was optional and as only 126 of the original 299 participants provided their contact information and further 38 did not participate in the follow-up study, this could result in a selection bias with potentially systematic differences in the sample. Also, because further 36 participants indicated that their loved one passed away, this study had an overall low study sample, indicating the need for further studies with larger samples. Due to the relatively small longitudinal samples, the analyses were able to detect medium-sized effects, but may not have been sensitive enough to identify small effects, therefore, statistically non-significant associations should be evaluated with caution. In addition, the number of predictors and covariates relative to the analytic sample size may have affected the stability of the regression estimates.

Moreover, only a small number of participants reported contact to any type of support institution, which may have limited statistical power and the stability of the regression estimates. Therefore, findings concerning support-use variables should be interpreted cautiously. Furthermore, participants were able to state that they used several types of support at the same time, which could have led to possible interaction effects. At the same time, after reviewing the data, we only found this in the case of a single participant who reported to have had regularly visited the general practitioner/specialist, the psychotherapist and the category “other” at the same time.

Moreover, pre-loss grief was defined more broadly without specifying the disease stage, treatment intent, treatment response, remission status, recurrence or transition to palliative care. Therefore, observed changes in pre-loss grief should be interpreted cautiously, as these factors may have significantly altered grief trajectories. Although subjective prognosis was assessed, a decrease in grief scores could reflect improvement in patients’ conditions, as treatments may have had a curative effect. Also, as the Caregiver Grief Scale was originally developed in dementia caregivers, some items may be interpreted differently by relatives of people with cancer, for example, items referring to the perceived absence of the loved one or loss of communication may be interpreted differently when the patient is still able to communicate, undergoing active treatment, or not in an end-of-life situation. Furthermore, although baseline pre-loss grief was controlled for and no baseline differences in psychological health were found, support use at T1 may still reflect help-seeking behavior, caregiving stress, or other unmeasured factors. Similarly, several other potential confounders and clinically relevant caregiving-related factors — such as relationship to the patient, cohabitation, primary caregiver status, caregiving burden, disease stage, treatment intent, palliative care involvement, patient functional status, caregivers’ mental health history, and previous bereavement experiences — were not assessed or included in the models, which may have resulted in residual confounding. Also, while completers and dropouts showed no significant differences in assessed variables, other important variables were not controlled for, which may result in some selectivity in the longitudinal sample.

Lastly, this study only focused on relatives of people with cancer, not including relatives of people with other illnesses such as dementia. Future research could investigate relatives of people with different illnesses and possibly compare their experiences of pre-loss grief.

## 6 Conclusion

This study demonstrates the change of pre-loss grief over a period of 1.5 years and indicates the association of various types of support on change of pre-loss grief. This study suggests that some aspects of grief before a loss, particularly emotional pain, may lessen over time, however, this finding should be interpreted cautiously given the stability of other grief dimensions and the lack of detailed information on patients’ clinical course. At the same time, because pre-loss grief seems to predict some negative health outcomes 1.5 years later, results suggest that high pre-loss grief may be relevant in care contexts. As results found the attendance of a psychotherapist to be associated with the reduction of pre-loss grief, these findings may inform future intervention research. However, attendance of any support institution was very low, so findings should be interpreted cautiously. To understand underlying mechanisms, further dismantling studies are needed.

## Data Availability

The raw data supporting the conclusions of this article will be made available by the authors, without undue reservation.
