# Supplementary material for: Change of pre-loss grief in relatives of cancer patients: a longitudinal study
Source: Front Psychol. 2026 Jul 16;17:1880352. doi: 10.3389/fpsyg.2026.1880352 (PMC13422434; doi:10.3389/fpsyg.2026.1880352)
Supplement: Supplementary file 1 [file Table_1.DOCX]

Supplementary Material. Differences between participants who completed follow-up and those who dropped out.

| *M (SD)* or *n* (%) | Follow-Up Completers with relative alive (*N*=52) | Dropouts (*N*=210) | *p*-value |
| --- | --- | --- | --- |
| Age | 38.67 (12.10) | 41.76 (12.34) | .41 ^a^ |
| Age of person with cancer | 47.69 (21.26) | 54.70 (19.97) | .16 ^a^ |
| Gender  Female  Male  Diverse | 46 (88.5)  6 (11.5)  0 (0.0) | 190 (90.5)  19 (9.0)  1 (.05) | .99 ^b^ |
| Gender of person with cancer  Female  Male  Diverse | 28 (53.8)  24 (46.2)  0 (0.0) | 114 (54.3)  95 (45.2)  1 (0.5) | 1.00 ^b^ |
| Subjective Prognosis | 44.92 (35.49) | 59.56 (37.69) | .07 ^a^ |
| School education |  |  | .97 ^b^ |
| Low | 1 (1.9) | 11 (5.4) |  |
| Medium | 16 (30.8) | 68 (33.1) |  |
| High | 35 (67.3) | 126 (61.5) |  |
| Diseased person is my  Child  Sibling  Parent  Partner  Friend  Other | 8 (15.4)  5 (9.6)  21 (40.4)  13 (25.0)  3 (5.8)  2 (3.8) | 24 (11.4)  15 (7.1)  87 (41.4)  54 (25.7)  6 (2.9)  24 (11.5) | .85 ^b^ |
| Religious (yes) | 36 (69.2) | 120 (57.4) | .47 ^b^ |
| Pre-loss grief, T1 | 3.45 (.68) | 3.46 (.79) | 1.00 ^a^ |
| Somatization, T1 | 9.02 (4.91) | 9.40 (5.60) | .97 ^a^ |
| Depression, T1 | 9.75 (6.72) | 9.70 (5.89) | 1.00 ^a^ |
| Anxiety, T1 | 11.04 (5.86) | 10.55 (5.39) | .91 ^a^ |
| Social support, T1 | 19.67 (4.42) | 18.84 (5.20) | .64 ^a^ |
| Contact to, T1  Support Group  Counselor  Psychiatrist  General Practitioner/Specialist  Psychotherapist  Other | 1 (1.9)  2 (3.8)  1 (1.9)  2 (3.8)  3 (5.8)  4 (8.3) | 12 (5.7)  11 (5.2)  7 (3.3)  19 (9.0)  23 (11.0)  6 (3.3) | .85 ^b^  1.00 ^b^  1.00 ^b^  .64 ^b^  .85 ^b^  .56 ^b^ |

^a^ two-tailored t-test, ^b^ χ² or Fisher’s exact test, ^c^ not including those with deceased relative at follow up.
